# The glass of milk half-empty? Dairy development and nutrition in low and middle income countries

**DOI:** 10.1016/j.foodpol.2023.102585

**Published:** 2024-01

**Authors:** Derek D. Headey, Harold Alderman, John Hoddinott, Sudha Narayanan

**Affiliations:** a The International Food Policy Research Institute (IFPRI), United States; b H.E. Babcock Professor of Food & Nutrition Economics and Policy Division of Nutritional Sciences, Applied Economics and Management, United States

**Keywords:** Dairy, Milk, Undernutrition, Stunting, Value chains, Nutrition education

## Abstract

Dairy products have an exceptionally rich nutrient profile and have long been promoted in high income countries to redress child malnutrition. But given all this potential, and the high burden of undernutrition in low- and middle-income countries (LMICs), why isn’t dairy consumption more actively promoted in the developing world? In this review we focus on a broadly defined concept of “dairy development” to include production, trade, marketing, regulation, and demand stimulation. We address three key questions. First, how strong is the evidence on the importance of dairy production and consumption for improving nutrition among young children in LMICs? Second, which regions have the lowest consumption of dairy products? Third, what are the supply- and demand-side challenges that prevent LMICs from expanding dairy consumption? We argue that although more nutrition- and consumer-oriented dairy development interventions have tremendous potential to redress undernutrition in LMICs, the pathways for achieving this development are highly context-specific: LMICs with significant agroecological potential for dairy production primarily require institutional solutions for the complex marketing challenges in perishable milk value chains; lower potential LMICs require consumer-oriented trade and industrial approaches to the sector’s development. And all dairy strategies require a stronger focus on cross-cutting issues of nutrition education and demand creation, food safety and quality, gender and inclusiveness, and environmental sustainability and resilience. We conclude our review by emphasizing important areas for research and policy expansion.

## Highlights

• Dairy products are highly nutritious, but under-consumed in LMICs.
• Dairy production and consumption strongly linked to reduced stunting risks in LMICs.
• Milk is expensive in LMICs, highly perishable and faces many supply-side challenges.
• Yet there are diverse dairy success stories based on production, but also imports.
• Dairy development needs to be nutrition-oriented, sustainable and context-specific.

## 1 Introduction

Of the hundreds of different foods produced, traded, processed, and consumed – including many that make vital contributions to health and nutrition – why does dairy warrant special attention for nutritional policy objectives?

First and foremost, consumption of dairy products is strongly associated with reductions in chronic undernutrition in early life, especially stunting, as papers in this collection of articles demonstrate (see below). The vast majority of infants and young children in low and middle income countries (LMICs) are fed monotonous diets with low intake of nutrient-rich fruits, vegetables and animal-sourced foods (ASFs) (Gatica-Domínguez et al., 2021). Poor diets are a root cause of undernutrition in early childhood, and undernutrition is astonishingly costly, accounting for almost half of deaths among children under 5 years of age (Black et al., 2013), poor schooling and cognitive outcomes and lower wages in adulthood (Hoddinott et al., 2013). Progress against stunting has been highly uneven, with only 55% of 194 countries on track to meet even one of nine World Health Assembly nutrition targets prior to the COVID-19 pandemic (Heidkamp et al. 2021). Growth in dairy consumption could improve diets and thereby accelerate progress towards nutrition targets, and simultaneously improve schooling and cognitive development outcomes.

Second, the glass is very much half-full: dairy consumption is very low in LMICs, with massive scope for growth. We ask why and dig into likely answers. Incomes are low in LMICs and dairy is often very expensive relative to staple foods (Headey and Alderman 2019). However, demand for dairy increases rapidly with income, and dairy products are widely perceived by caregivers as nutritious child-friendly foods. If dairy products can be made more affordable – through production, trade, or other food policies – then rapid growth in consumption can be expected follow. If nutrition education, school feeding, or social protection policies can strengthen demand for healthy dairy products for the poorest populations and for young children in particular (and curb demand for unhealthy dairy products), then the nutritional impacts can be further amplified.

Third, many poor smallholders and even landless rural households in LMICs own dairy cows (FAO, GDP, and IFCN, 2018), and dairy production is often (but not always) a responsibility of women. However, women and other disadvantaged groups are often marginalized from dairy marketing decisions and income streams, and even from dairy development interventions and institutions. There are, however, examples of inclusive dairy development initiatives that are pro-poor, gender-sensitive and nutrition-sensitive, as we review below.

Fourth, dairy is a relatively unique product with complex economic characteristics. Fresh milk is highly perishable and costly to trade long distances; conversely, powdered milk is highly storable and tradable. Dairy sectors can be highly dualistic in LMICs, with large informal sectors engaged in localized trade and exceptionally high rates of auto-consumption of dairy among dairy-producing households, and more formal sectors engaged in processing, packaging, advertising, and long-distance trade. Milk’s perishability creates many challenges in its domestic value chains. In rural LMIC settings characterized by large numbers of dispersed smallholders producing a highly perishable product, poor access to markets is often the binding constraint on technology adoption, and hence the development of the sector. Innovative institutional arrangements are therefore required to connect smallholders to some kind of marketing body, be it a cooperative, a dairy hub, a milk collection center (MCC), a private firm contracting individual farmers, or a mega-firm hiring farmers or even leasing their cows to produce milk. Those market connections can then indirectly or directly provide the impetus for farmers to adopt improved technologies, especially improved dairy breeds. Few other food sectors in LMICs have such a diverse array of institutional arrangements in their value chains, with commensurate diversity in the success or failures of these arrangements. Yet despite many challenges, the growing number of diverse dairy development success stories in LMICs offers hope of replication or adaptation in other LMIC settings.

These considerations collectively motivate this review in which we flesh out these arguments in more detail, drawing on both the existing literature and the articles published in this special collection. We note that both the special collection and this review focus on cow’s milk, though many of the issues we discuss are pertinent to milk and dairy products from goats, sheep, camels, and certain buffalo, all of which produce highly nutritious products but also highly perishable (FAO, 2013a, FAO, 2013b). The remainder of the study focuses on: (1) the evidence on the nutritional importance of dairy; (2) documenting dairy consumption gaps across and within LMICs; and (3) explaining these gaps in terms of both supply- and demand-side constraints. Finally, we conclude the paper with suggestions for further research, and a call for food policymakers to closely heed the evidence in this body of research and invest more to scale up dairy consumption in LMICs - especially among children and other nutritionally vulnerable groups – through innovative, systematic and context-specific interventions and institutional arrangements.

## 2 Why the glass should be full: Evidence on the nutritional importance of dairy

### 2.1 Nutritional properties of dairy products

Dairy products have a range of nutritional and physical characteristics that make them an almost ideal complementary food for infants and young children, but also nutritionally beneficial for older children and adults, provided they can tolerate some amount of lactose.

First, dairy is rich in macronutrients, energy, fats, and high-quality protein, all of which could be critical constraints for children from highly food insecure households. Cow’s milk has a higher digestibility-corrected amino acid score than any other food and is particularly efficacious at closing amino acid gaps in the cassava- and cereal-heavy diets prevalent in Africa and Asia, which are often deficient in lysine (FAO/WHO/UNU, 2007).

Second, dairy is unique in stimulating plasma insulin-like growth factor 1 (IGF-1), a growth hormone that acts to increase the uptake of amino acids (FAO, 2013a, FAO, 2013b). IGF-1 may help explain why dairy products appear especially efficacious in stimulating linear growth (de Beer, 2012). Neuroscientists have also linked IGF-1 to central nervous system development and maturation, as well as learning and memory (Dyer et al., 2016). Some authors also claim there is evidence that dairy products may stimulate weight gain and muscle accretion in wasted children (Grenov and Michaelsen, 2018, Michaelsen, 2013).

Third, dairy has a rich micronutrient profile. Dairy is best known for high levels of calcium, which contributes to bone length and strength and may prevent nutritional rickets and stunting in African and Asian populations (FAO, 2013b). Processed dairy products can also be fortified with vitamin D. Dairy is also rich in other critical micronutrients, including vitamin A and B12, as well as potassium, magnesium, and phosphorus (Dror and Allen, 2014).

Finally, the sheer density of multiple macro- and micronutrients in dairy products – as well as their taste and similarity to human breastmilk – make them ideal for infants and young children with small stomachs incapable of consuming large quantities of foods with low density of micronutrients. Dairy products also have very short preparation times, making them convenient for busy caregivers (though cold storage is required for most dairy products).

### 2.2 Impacts of dairy consumption on linear growth and stunting

Although the potential nutritional benefits of dairy have been recognized since the introduction of school milk programs in the United Kingdom in the 1920s (Leighton and Clark, 1929), most systematic reviews of the evidence on dairy consumption and child growth pertain to school age children, often in high income countries (de Beer, 2012, Iannotti et al., 2013). However, there is little experimental evidence assessing dairy’s impact on child nutrition outcomes in younger children in LMICs,^1^ which has motivated a relatively recent literature using observational or quasi-experimental studies to assess associations between HAZ/stunting and ASF consumption in LMICs.

One approach uses large multi-country observational studies, drawing on the Demographic Health Surveys (DHS), to analyze the association between stunting and ASF consumption in the past 24 h among children 6–23 months of age. Headey et al. (2018) and Herber et al. (2020) both find that dairy consumption significant reduces the risk of stunting, and Headey et al. (2018) find that the magnitude of the risk reduction is significantly larger for dairy than for other ASFs.

A second approach uses quasi-experimental methods to assess linkages between cattle ownership, dairy consumption and HAZ/stunting. Here we find consistent findings among studies conducted in Rwanda (Rawlins et al., 2014), Ethiopia (Hoddinott et al., 2015), Uganda (Kabunga et al., 2017) and Bangladesh (Choudhury and Headey, 2018). Remarkably, almost all of these find that dairy cow ownership or recent dairy production predicts roughly a 0.5 standard deviation increase in HAZ^2^; a strong association similar to child HAZ differences between rich and poor households, for example.

Three papers published as part of this collection strengthen this evidence base. Unlike the cross-sectional studies above, Zoniaina et al. (2023) use a nine-round panel survey from central Madagascar to examine dairy consumption/production and child growth longitudinally. With household fixed effects models, they find that lagged milk production is a strong predictor of child HAZ. In an unusually rich multi-round survey from Bangladesh, Bakhtiar and Hoddinott (2023) also re-affirm the strong association between HAZ and dairy production (with results again close to the 0.5 standard deviation “effect” size), and extend the literature to examine whether male and female ownership of dairy cows influences dairy-HAZ associations (they might). Finally, Haile and Headey (2023) offer novel “macro” evidence by employing a large cross-country panel to show that that increases in per capita milk consumption are a robust predictor of national reductions in stunting prevalence; indeed, at that very macro level, dairy consumption growth is the *only* food group that predicts stunting reduction. Thus, a wide range of evidence provides strong and consistent support to the hypothesis that dairy consumption is very effective in promoting linear growth and stunting reduction in young children.^3^

### 2.3 Impacts of dairy consumption on other nutrition and health outcomes in children and adults

Higher dairy intake reduces micronutrient deficiencies in specific demographic groups (McGill et al., 2008, Moore et al., 2012, Weinberg et al., 2004). In LMICs specifically, dairy intake has been shown to increase intakes of calcium, vitamins A, D and B12; e.g., in pre-school children in Indonesia (Sunardi et al., 2022), in school age children in India (Kumar et al., 2021), and among lacto-vegetarian young adults in India (Naik et al., 2013). In this special collection, Ecker and Pauw (2023) simulate the impacts of a dairy price subsidy on household micronutrient consumption gaps in Uganda and Kenya. They show that dairy price subsidies are quite an effective tool for closing nutrient adequacy gaps in calcium, riboflavin, and vitamin B12; indeed, they are more effective than income transfers for these specific micronutrients.

Concerns are sometimes raised that dairy contributes to childhood obesity, but actually the opposite seems true, with dairy products (not artificially sweetened) being linked with weight loss among higher income individuals otherwise at risk of obesity, while in the longer term dairy consumption is associated with a tall-but-thin phenotype (Dougkas et al., 2019, Kang et al., 2019, Snijder et al., 2007, Thorning et al., 2016, Visioli and Strata, 2014).^4^ The fact that dairy products can have relatively high fat content but not lead to obesity (and even reduce risks of obesity) is surprising on the surface, but may be related to dairy’s high calcium content (Zemel, 2004), its ability to satiate appetite, to promote healthy gut microbiota, and to achieve fat and energy balance (Dougkas et al., 2019). Less surprising is that there is also a beneficial effect of milk and dairy intake on bone mineral density (de Lamas et al., 2019, Rizzoli, 2022, van den Heuvel and Steijns, 2018, Wallace et al., 2021).

A more recent concern is that milk produced from contaminated feed can contain aflatoxin M1 (AFM1), a metabolite of the carcinogenic aflatoxin B1 (AFB1), a known carcinogen. However, a review of evidence in this special collection indicates that there is no significant increase in liver cancers from AFM1 contamination in dairy since AFM1 is much less carcinogenic than AFB1 (Saha Turna et al., 2022). A key policy implication of that study is that food safety regulations on AFM1 in dairy may be overly stringent.

A less dramatic concern is lactose intolerance. Adult populations in many parts of the world – including large segments of Africa, Asia and Latin America – lack an enzyme to digest lactose, the key sugar in milk (Anguita-Ruiz et al., 2020; Heyman, 2006; Storhaug et al., 2017). Lactose intolerance can lead to malabsorption and discomfort but some populations with high rates of intolerance, such as South Indians and Ashkenazi Jews, regularly include dairy products in their diets. Moreover, most individuals who lack the enzyme to break down lactose can tolerate 240 g of milk with minimal symptoms (Bhatnagar and Aggarwal, 2007). Enzymes to digest lactose are present in all infants but the enzyme declines as children age. Lactose intolerance is uncommon before 2 to 3 years of age in all populations (Heyman, 2006), implying that dairy products are generally highly suitable as complementary foods give their high nutrient density.

One area requiring more evidence is assessments of the impact of dairy consumption on cognitive development, especially in younger children. There are biological mechanisms that theoretically link dairy consumption to brain development, and studies showing impacts of dairy supplementation in school feeding programs on cognitive test scores among older children (Lee et al., 2018), but there is little evidence yet on impacts among younger children.

## 3 The glass half full: The global divergence of dairy consumption

### 3.1 Average dairy consumption patterns by region

**Fig. 1:**
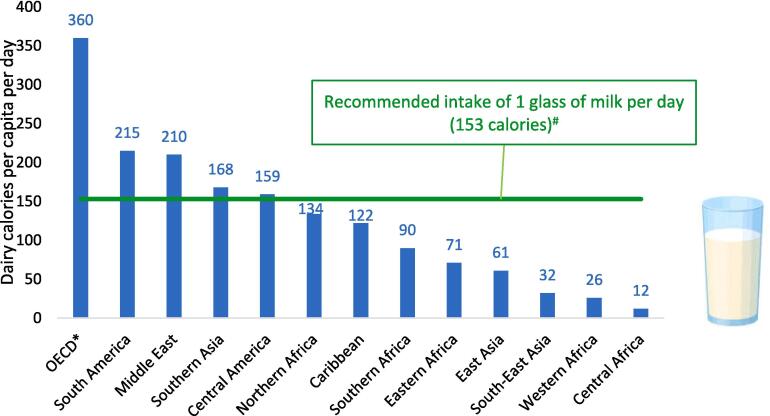
Estimated daily supply of dairy calories per capita against the recommended intake of at least 1 glass per day (153 calories), by region). Notes: Authors’ construction from the FAO Food Balance Sheets for 2020 (FAO, 2023), while the recommended intake is taken from the midpoint reference value from the EAT-Lancet reference diet (Willett et al., 2019). *OECD refers to high-income OECD countries only.

Despite the evidence above, the full potential of dairy to redress the global burden of malnutrition is unrealized. Fig. 1 reports estimates of the average daily supply of dairy calories per capita by region against the recommended intake of 153 calories per day (roughly one glass of milk) from the EAT-Lancet reference intake (Willett et al., 2019). Note that these FAO Food Balance Sheet data include consumption of all dairy products, including obviously unhealthy foods like ice cream, sugar sweetened dairy beverages and South Asian dairy sweets.

In per capita terms, high income OECD countries consume over twice the recommended levels (360 calories per day), while populations in Latin America, Western Asia, and North Africa consume roughly the recommended amount, at least on average. South Asians reach the 153 calorie/day target, on average, these supply data likely mask considerable inequality within the region and within countries like India where only half of young children consume dairy on a daily basis (see below). Unlike South Asia, most of East and South-East Asia has weak dairy traditions and consumption is low though often growing rapidly. South-East Asia’s supply levels are just 32 calories per day, while East Asia’s is around 61 calories per day; though several decades ago these regions consumed almost no dairy. Southern Africa’s dairy supply is around two-thirds the recommended daily intake level at 90 calories, although this is dominated by above-average supply levels in South Africa. In East Africa, which has strong dairy traditions and cattle ownership is widespread, dairy supply still only reaches 71 calories per day, pointing to low productivity and very limited imports (more on this below, also). Even so, East Africa’s average consumption levels are double that of West Africa (32 calories per day) and five times that of Central Africa (a paltry 12 calories per day).

### 3.2 Dairy consumption patterns among infants and young children by region

The FAO data in Fig. 1 do not offer information on consumption inequality within countries, or consumption among nutritionally vulnerable infants and young children. Hence, Table 1 uses DHS data on whether a child 6–23 months of age consumed dairy (excluding infant formula) or another ASF in the past 24 h, using simple yes/no answers without quantitative information.^5^ Despite that caveat, these child-level data add considerable nuance to the per capita consumption patterns observed in Fig. 1.^6^

**Table 1:** Consumption of dairy and non-dairy animal sourced in the past 24 h by children 6–23 months of age in developing regions and countries.

**Regions & Example Countries (N = number of countries per region)**		**Dairy**	**Other animal sourced foods**
***Eastern Europe & Central Asia***	***Average (N = 5)***	***56%***	***58%***
	Moldova	54%	75%
	Armenia	69%	51%
	Tajikistan	50%	35%

***Latin America & Caribbean***	***Average (N = 6)***	***48%***	***67%***
	Dominican Rep.	75%	65%
	Peru	56%	85%
	Guatemala	35%	66%

***Middle-East & North Africa***	***Average (N = 3)***	***52%***	***47%***
	Egypt	63%	52%
	Jordan	42%	56%
	Yemen	51%	33%

***South Asia***	***Average (N = 4)***	***42%***	***41%***
	Pakistan	53%	38%
	India	41%	21%
	Bangladesh	25%	69%

***South-East Asia***	***Average (N = 4)***	***25%***	***68%***
	Vietnam	55%	89%
	Cambodia	16%	82%
	Myanmar	14%	58%

***East African highlands***	***Average (N = 3)***	***37%***	***28%***
	Kenya	51%	32%
	Ethiopia	32%	13%
	Uganda	28%	39%

***Southern Africa***	***Average (N = 9)***	***20%***	***44%***
	South Africa	32%	58%
	Tanzania	22%	36%
	Malawi	9%	38%

***West and Central Africa***	***Average (N = 22)***	***17%***	***47%***
	Senegal	34%	45%
	Nigeria	24%	44%
	Congo, Dem. Rep.	6%	52%

Notes: “Average” refers to unweighted averages across countries. Data are from the DHS (ICF-International, 2022) for the most recent survey over 2008–2021 time period, while 2014 Vietnam data are from UNICEF (2022).

Table 1 demonstrates marked differences within and across regions. Roughly 50–60% of children in Latin America, the Middle East and North Africa, Eastern Europe and Central Asia consumed dairy in the past 24 h. In these regions dairy is often as widely consumed as all other ASFs together. ASF consumption is 42% in South Asia, which is high relative to the region’s relatively low-income levels. However, in South-East Asia just 25% of children consumed dairy. In three East African highland countries with strong dairy traditions, 37% of children consumed dairy, but in 9 Southern African countries this falls to just 20%, and in 22 West and Central African countries it falls further to 17%.

Within regions, however, there is also marked inequality in dairy consumption. Just 35% of children consumed dairy in Guatemala, a country where roughly half of children are stunted, whereas 75% of children consumed dairy in the Dominican Republic where just 7% of children are stunted.

In South Asia, India, Pakistan, and Nepal have relatively high dairy consumption, but in Bangladesh (where fish is far more common than dairy) just 25% of children consumed dairy in the past 24 h compared to 69% consuming other ASFs. We note the special importance of dairy in India, where one third of the population are lacto-vegetarians who consume dairy but typically no other ASFs (Headey and Palloni, 2020). Indeed, it is striking that while 41% of Indian children consumed dairy in the past 24 h, just 21% consumed any other ASF. This suggests, also, that non-dairy ASF consumption is unusually low in India, even among the non-vegetarian population (Headey and Palloni, 2020), and that dairy plays an exceptionally important role in adding high quality protein, fat and various micronutrients to Indian diets.

Within Africa, half of Kenyan children consumed dairy in the past 24 h, compared to less than one third of children in Ethiopia and Uganda, despite widespread cattle ownership in all three “highland“ countries. In the rest of sub-Saharan Africa, dairy consumption among children is very low, including in more populous countries such as Nigeria (24%) and the Democratic Republic of Congo (DRC) (just 6%). It is notable that stunting rates are very high in Nigeria (37%) and the DRC (43%) compared to Kenya (26%) and Uganda (28%).

## 4 Why is the glass half full? Explaining low dairy consumption in LMICs

Why is dairy so under-consumed in LMICs?

**Fig. 2:**
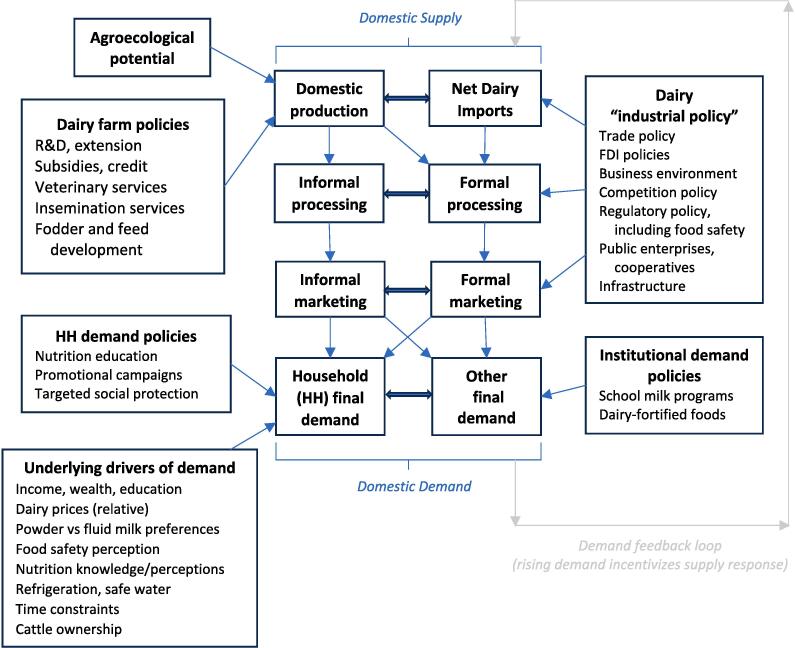
A stylized framework for understanding dairy supply and demand in the developing world.

Answering that question requires a framework to understand the complexities of supply and demand of dairy products, which we try to encapsulate in Fig. 2. Some quite unique and interconnected features to consider in LMIC dairy sectors are:

(1) Strong associations between climate conditions and dairy productivity (Herrero et al., 2016), with dairy productivity being much higher in more temperate conditions;
(2) Modest economies of scale and high degrees of labor intensity in dairy compared to other ASF sectors, such as poultry (Narrod et al., 2007);
(3) The exceptionally high perishability of dairy products in the absence of refrigeration or processing (unlike other foods, unrefrigerated dairy products can spoil in a matter of hours);
(4) Exceptionally strong linkages between household production and consumption of milk because of perishability and the availability of “evening milk”^7^ supplies (Choudhury and Headey, 2018, Hoddinott et al., 2015, Sibhatu and Qaim, 2017);
(5) Robust persistence of informal markets and potential market segmentation of formal and informal markets (Narayanan et al., 2023);
(6) Acute marketing coordination challenges for collecting perishable milk from dispersed rural smallholders (Van Campenhout et al., 2021, Vandercasteelen et al., 2021);
(7) High prices of dairy products, especially in countries with low levels of production or dependence on imports (Headey and Alderman, 2019);
(8) Heterogenous consumer preferences for fresh or powdered milk, and sometimes even for unpasteurized milk (Blackmore et al., 2022, Grace et al., 2020), as well as potentially strong cultural norms around milk consumption;
(9) Sizable food safety risks with unpasteurized milk or adulterated dairy products (Grace et al., 2020), making dairy a credence good;
(10) Large heterogeneity in whether dairy is a traditional part of the diet (e.g. India, Ethiopia, Kenya) or relatively novel (China, Vietnam, Thailand), and where demand may be changing rapidly due to economic growth, globalization of diets and other factors; and
(11) Unusually strong consumer perceptions that dairy is a nutritionally important food for children, which is often reinforced by public health messaging (Smitasiri and Chotiboriboon, 2003).

In this section we elaborate on these unique features of dairy markets further and use the framework in Fig. 2 to consider the numerous challenges to dairy development in LMICs, as well as important LMIC success stories.

### 4.1 Dairy production challenges in LMICs

Dairy production potential is heavily influenced by agroecological conditions, with dairy yields substantially higher in more temperate climates that support high-yielding breeds. In addition, heat stress affects fertility and milk yields and potentially the quality of feed (Herrero et al., 2016, Robinson et al., 2011). Climatic factors are also strongly associated with livestock disease and pests, such as tsetse fly in Africa (Alsan, 2015, Pingali et al., 1987). Thus, the impacts of climate on dairy production operates both through the intensive margin (yields) and the extensive margin (ownership and herd size), by which farmers do not invest much in dairy cattle ownership because of the high costs and sizable risks involved.

Table 2 provides some empirical basis for this claim by reporting regional averages (unweighted) of the percentage of rural households owning cattle (the extensive margin)^8^ and regional averages of dairy yields (the intensive margin). The more temperate climates of South America, Central Asia and the East African highlands are conducive to cattle ownership, with roughly half of all rural households owning cattle on average, reaching as high as 80% in Ethiopia, where high-altitude plateaus and arid lowlands inhibit tsetse fly and several other livestock diseases. Likewise, arid areas, such as the Sahel, have relatively high rates of cattle ownership (50% on average), whereas the humid climates of coastal Western Africa and Central Africa – which are ideal for tsetse fly – have very low rates of cattle ownership (16% and 12% respectively), with just 2% of households in the Democratic Republic of Congo owning cattle. Cattle ownership is moderate in Southern Africa (30%), which has a mix of humid, temperate, and arid climates. In South Asia, around half of rural households own cattle or buffaloes, but this falls to just 27% in South-East Asia.

**Table 2:** Indicators of constraints on the extensive margin (cattle ownership) and intensive margin (dairy yields) across regions.

	**Extensive margin**	**Intensive margin (dairy yields)**	
	**Cattle ownership ^a^ (% of rural households)**	**Dairy yields ^b^ (litres/animal)**	**Dairy yields relative to “dairy frontier”**
North America, Europe, Australasia (“dairy frontier”)^c^	NA	6,219	100%

South America	39% ^d^	1,902	31%
Central America & Caribbean	14% ^d^	2,074	33%

Central Asia	58% ^d^	2,083	33%
Middle East	NA	513	8%
North Africa	NA	199	3%

South Asia	47%	1,097	18%
South-east Asia	27%	307	5%

Southern Africa	33%	47	1%
Eastern Africa	50%	293	5%
Central Africa	12%	137	2%
West Africa, coastal	16%	56	1%
Sahel	50%	24	1%

Notes: a. Cattle ownership among rural households is sourced from the DHS (ICF-International, 2022). b. Dairy yields are sourced from the FAO (FAO, 2023). c. “Dairy frontier countries” are defined as countries with high dairy yields and temperate climates, including the US, Canada, Western European countries and Australia and New Zealand. Estimates for these samples are indicative only because of small DHS samples sizes for these regions.

Turning to the intensive margin, the FAO estimates that the average country in Europe, North America, and Australasia – which can be thought of as the “dairy frontier” – has milk yields that exceed 6,000 L per year for a typical dairy cow. A typical commercial dairy cow in New Zealand, for example, can produce around 28 L per day for 10 months of the year, enough to feed 120 children a glass of milk per day. In the relatively temperate climates of Latin America and Central Asia, dairy cows produce around 2000 L per year, one third of the frontier countries. In South Asia yields are just 18% of the frontier, but they are as low as 5% in South-East Asia. In sub-Saharan Africa estimated yields are between 1 and 5% of the frontier countries. Even in Kenya, where conditions are relatively favorable for dairy production, official yield estimates are just 20% of the levels of South Africa.^9^

Improving dairy productivity is highly context-specific, contingent upon climate, feed availability, market access, and other factors. That said, it is possible to generalize about some common constraints in LMICs (Herrero et al., 2016, Thornton, 2010). On a purely technical level (abstracting from market incentives to adopt new technologies), productivity gains almost invariably require improving the genetic composition of LMIC dairy herds, including significant upfront investment in either imported breeds or cross-breeds (Thornton, 2010). However, realizing productivity gains from improved breeds requires a host of other inputs, including efficient veterinary services, insemination services, access to affordable improved feed, and improved herd management practices (Herrero et al., 2016, Thornton, 2010). Still, pushing ahead with these technologies is fraught with risk if farmers have insufficient access to dairy marketing channels.

### 4.2 Dairy value chain challenges in LMICs

The hyper-perishability of milk makes efficient processing, storage, transport, and marketing acutely important for commercialization, and indeed for adoption of the kinds of technologies described above. Solving these “market access” challenges in rural settings, where many smallholders are dispersed and hard to reach, is arguably the most critical step in the development of domestic dairy sectors in LMICs. However, different countries and different private and public firms have found quite diverse means of solving these challenges.

Ethiopia exemplifies these marketing challenges and remains relatively unsuccessful at developing its dairy sector as extensively as the government and the industry would like. Although cattle ownership is high and dairy is a traditional food, yields are just 5% of “dairy frontier” levels, and most smallholders are remote from markets and use cattle for multiple purposes in addition to dairy (Behnke, 2010). In rural areas milk is much less marketed than other foods: an astonishing 80% of milk quantities consumed in rural Ethiopia is sourced from a household’s own farm (Sibhatu and Qaim, 2017), a proportion far higher than other food group. Moreover, one survey found that 55% of rural markets in Ethiopia did not even sell any dairy products, compared to 90–100% for other food groups (Headey et al., 2019).

Such low marketing of milk in rural Ethiopia suggests that the fundamental market access challenge for dairy has not been solved, although a recent study on the Addis Ababa dairy market illustrates the transformational effects of improved market access. Addis Ababa’s rapid income growth and expansion to almost 6 million people have to led to increased demand for dairy, and the number of dairy processing firms in Ethiopia tripled over 2007–2017 (Minten et al., 2020). However, almost one-third of all liquid milk in Addis Ababa is sourced from the *urban* farm sector, not the rural sector. Farmers not in close proximity to Ethiopian cities have lower adoption of modern technologies and limited access to dairy services, with the result that yields have stagnated (Minten et al., 2020, Vandercasteelen et al., 2021). Indeed, with each additional hour of travel time to Addis Ababa, farmers’ milk productivity per cow falls by 26% (Vandercasteelen et al., 2021).

The most common approach to solving market access problems in smallholder settings has been the promotion of dairy cooperative models, which have a long history in “dairy frontier” countries such as New Zealand (extending back to 1871).^10^ India’s Anand Pattern cooperative model is the most well-known success story in LMICs, and has been widely adapted elsewhere, albeit with varying success. Originating in Gujarat state in the late 1940s, the Anand Pattern cooperative model is an integrated three-tiered structure that procures, processes, and markets dairy produce. At the village level, dairy cooperative societies (DCS) are formed by milk producers, and any producer can become a member by buying a share and committing to sell milk only to the DCS milk collection center (MCC). Each member's milk is tested for quality with payments contingent on testing outcomes. At the end of each year, a portion of the DCS profits is used to pay each member a bonus based on the quantity of milk procured. At the district level, these cooperatives form a union that buys, processes, and markets all DCS milk, and most unions provide the inputs for both milk production and the cooperatives' business. Finally, State Federations are responsible for collecting and marketing fluid milk and products of member unions, and some federations also manufacture feed and support other union activities.

The description above is highly stylized for such a large and diverse country, but an analysis in this special collection of administrative data on Indian dairy cooperatives over a 20-year period shows a gradual development path that has moved from collection to marketing and diversified processing (Dervillé et al., 2023). The authors identify a transition between scaling out (improving market access for farmers) and scaling up (systemic change in the dairy activity, such as processing). They show that scaling up is most advanced in western Indian states with a long history of cooperatives and strong dairy traditions, but is much less advanced in Eastern and Northeastern India, with South Asia somewhere in the middle.

Cooperatives in India are broadly judged to have been successful in connecting smallholders with markets in a pro-poor fashion (Alderman, 1987, Candler and Kumar, 1998, Kumar et al., 2018). Kumar et al. (2018) show that membership in cooperatives in Bihar state has positive impacts on milk yields, profits, and adoption food safety measures. However, since the 1990s commercial sector mega-firms have expanded rapidly, most of whom have replicated the structure of local MCCs and local chilling units that were the backbone of the Anand Pattern (Burkitbayeva et al., 2023). These private firms have been a key driver of productivity growth in India, despite the persistent dominance of the informal sector in aggregate milk supplies. In this virtual collection, Kumar et al. (2019) use a national survey to find that integration with modern dairy value chain in India has a positive and significant impact on dairy household’s net returns and consumption expenditures.

In other countries, dairy cooperatives have had more mixed success. In Ethiopia there are only a handful of successful cooperatives despite much government support (Chagwiza et al., 2016, Gebreyohanes et al., 2021). Cited problems are heavy government oversight, historical mistrust of socialist cooperatives, low management capacity and low levels of services, low prices and – relatedly to all of these problems – frequent side-selling outside of the cooperative. Side-selling is also a concern in Kenya where milk cooperative members side-sell when facing uninsured health emergencies or when cash on hand is low (see Geng et al. (2023) in the virtual collection). It is also plausible to conjecture that promoting cooperatives is insufficient without simultaneous investments in dairy market infrastructure, especially MCCs with cooling facilities to provide the first point of entry in the dairy cold chain. In Rwanda, the government has simultaneously invested in MCCs in addition to promoting cooperatives, and some MCCs are run by cooperatives while others are privately owned (De Vries et al., 2020). In recent years, the Rwandan government has tried to mandate that all milk be sold through MCCs to formalize the sector and improve food safety, since MCCs have milk testing facilities. However, despite government directives, farmers still sell a significant portion of their milk to traders or local restaurants, although farmers in dairy cooperatives are more likely to sell to MCCs (see Habiyaremye et al. (2023) in this special collection).

Uganda’s dairy sector development has recently been more private sector focused. The government did promote dairy farmers to unite in cooperatives and set up their own milk collection centers, but also facilitated international trade relationships through tax breaks and free land allocations for dairy, and inviting foreign direct investment (FDI) into dairy processing capacity (Van Campenhout et al., 2021). Some foreign investors were attracted by low production costs in Uganda but they were also interested due to problems for their operations in other countries such as Kenya. Private sector investment has taken off. In a just a few short years foreign firms have become the major source of dairy processing, and production and export growth has expanded rapidly, especially in the South-West, resulting in Uganda switching from being a net dairy importer to a net exporter. This has resulted in rapid growth in the number of MCCs but also expansion in the services they provide. Van Campenhout et al. (2021) report that over two-thirds of surveyed MCCs in south-western Uganda stated that they provided advances to their clients, one half of the milk collection centers provide training on milk hygiene, and 45% of centers also provide aluminum milk cans to their clients, while many also supplied veterinary medicines and vaccinations (Van Campenhout et al., 2021).

In other LMICs, private contract farming is the dominant form of value chain organization (though co-existing with cooperatives). In some LMICs, dairy mega-firms have emerged, such as *Vinamilk* in Vietnam, which simultaneously procures milk powder through imports, and fresh milk through their own farms (i.e., vertical integration), through contract farming with individual farmers, and through contracts with cooperatives. Another large Vietnamese firm solves the coordination problem by organizing all milk production activities over a concentrated area of 1600 ha, contracting 565 farmers, providing credit and insurance, organizing input supplies, and milk collection, and processing through contract workers (Huang et al., 2023). In China, similar “dairy development parks” broadly follow the cluster model used in other sectors of Chinese industrial strategy, by facilitating processors to set up community-based “Dairy Parks” where smallholders keep and milk their cows, with the parks financed either by processors, local authorities or smallholders themselves (FAO, 2008). One company, *Dairy United*, leases dairy cows from local farmers, giving the firm access to a key asset without up-front investment, and letting the firm grow its own dairy herds with newborn heifers to achieve scale economies (Wang et al., 2015). In return, farmers receive fixed payments biannually but relinquish control rights. Foreign investment has also played an important role in dairy development in East Asian countries, though its role in the transformation of marketing institutions and technology upgrading is still not well documented.^11^

In summary, solving the marketing challenges of hyper-perishable fresh milk is critical for promoting domestic dairy production. The most effective solutions to dairy marketing challenges are likely quite context-specific, depending on local institutional, geographic and economic conditions.

### 4.3 Dairy trade challenges (and opportunities) in LMICs

Although fresh dairy products are highly perishable, powdered milk products are non-perishable and relatively cheap to import over long distances.^12^ Powdered milk can serve as either a retail product to be reconstituted by consumers themselves, or as an intermediate input into industrial reconstitution. Since some dairy products are highly tradable, and dairy seems in high demand, why don’t countries with low production potential simply import dairy products?

There are several possible answers to this complex question. First, many LMICs impose high tariff and non-tariff barriers on dairy products while simultaneously not producing sufficient supplies from their own sector, resulting in poor availability and high prices. India has a 35% tariff barrier, but also requires importers to have veterinary health certificates. Bangladesh – with far less domestic production potential than India – imposes a 25% tariff on most dairy products. In one recent gravity model analysis of OECD dairy trade with the rest of the world, memberships in trade agreements were an important determinant of dairy trade along with market size and the quality of government institutions (Kondaridze and Luckstead, 2023). A study in this special collection found that dairy tariff rates were positively associated with dairy prices, negatively associated with dairy consumption and positively associated with stunting rates (Liu et al., 2023). That suggests that LMICs may have trade regimes that protect domestic producers from competition from imports, but which harm consumers and prevent progress on child nutrition.^13^

Second, dairy is also subject to some degree of natural protection if consumers have strong preferences towards fresh local milk over powdered milk or other long-life milk products. That preference indeed appears to be prevalent in countries with strong dairy traditions (e.g. Kenya), and may only apply to fresh milk, with powder milk imports still being used for other dairy products, such as production of Bengali sweets in Bangladesh.

A third explanation is weak or stagnant demand for imports. Although dairy is often an aspirational good with high income elasticities (Colen et al., 2018, Ecker and Pauw, 2023), a combination of low incomes levels, high dairy prices, lack of household refrigeration (Heard et al., 2020), and food safety issues (including poor water quality) can hinder growth in demand for imported dairy products (Headey, 2023), as can macroeconomic barriers like foreign exchange constraints.

While more research is needed on dairy trade and industrialization strategies, Table 3 offers some insight into the growth of total supply of dairy products between 1990 and 94 and 2015–19 in some selected countries of interest. Total supply is equal to production plus net imports (all in per capita terms), and we also measure the “nutritional adequacy of supply” as supply per capita relative to the 250 g per day recommended by the EAT-Lancet diet (Willett et al., 2019).^14^ The LMICs selected are a mix of low and moderate production potential, and weak/moderate or strong dairy traditions, and all countries experienced reasonably strong income growth (and very rapid growth in the case of China and Vietnam).

**Table 3:** Trends in dairy production, imports and exports (grams per day) and the nutritional adequacy of supplies relative to the EAT-Lancet recommendations in selected LMICs over 1990–94 to 2015–19.

**Country**	**Production potential**	**Indicators**	**1990**–**94**	**2015**–**19**	**Change**
Bangladesh	Low	Production per capita (grams/day)	33.3	48.6	15.3
		Imports per capita (grams/day)	5.2	5.3	0.1
		Exports per capita (grams/day)	0.0	0.0	0.0
		Nutritional adequacy of supply^a^	16%	23%	7 points

China	Moderate	Production per capita (grams/day)	15.1	64.3	49.3
		Imports per capita (grams/day)	2.8	33.3	30.5
		Exports per capita (grams/day)	0.4	0.6	0.2
		Nutritional adequacy of supply^a^	7%	41%	34 points

Ghana	Low	Production per capita (grams/day)	4.0	4.2	0.2
		Imports per capita (grams/day)	6.3	15.6	9.2
		Exports per capita (grams/day)	0.0	1.1	1.1
		Nutritional adequacy of supply^a^	4%	8%	4 points

India	Moderate	Production per capita (grams/day)	146.3	301.9	155.5
		Imports per capita (grams/day)	0.1	0.0	0.0
		Exports per capita (grams/day)	0.1	2.8	2.8
		Nutritional adequacy of supply^a^	62%	127%	65 points

Thailand	Low	Production per capita (grams/day)	7.1	43.9	36.7
		Imports per capita (grams/day)	41.6	83.9	42.3
		Exports per capita (grams/day)	2.4	9.5	7.1
		Nutritional adequacy of supply^a^	20%	50%	30 points

Uganda	Moderate	Production per capita (grams/day)	62.5	106.1	43.6
		Imports per capita (grams/day)	1.3	0.1	−1.1
		Exports per capita (grams/day)	0.1	5.2	5.2
		Nutritional adequacy of supply^a^	27%	43%	16 points

Viet Nam	Low	Production per capita (grams/day)	2.3	20.1	17.8
		Imports per capita (grams/day)	6.3	109.1	102.8
		Exports per capita (grams/day)	0.0	1.5	1.5
		Nutritional adequacy of supply^a^	4%	54%	50 points

Notes: Authors’ construction from FAO Food Balance Sheets (FAO 2023). a. Nutritional adequacy of supply is the ratio of total supply per capita relative to the 250 g reference intake of the EAT-Lancet report (Willett et al., 2019), adjusted for demographic differences across countries using scaling factors from Headey et al. (2023).

What kinds of patterns and trends do we observe in these production and net import indicators?

There are a diverse range of success stories. India saw a doubling of total dairy supply driven entirely by domestic production growth (with trade barriers likely assisting domestic dairy development), but East and South-East Asian success stories (China, Vietnam, Thailand) have followed a more agro-industrial model relying on both production growth and growth in imports (Ava, 2014). China relies on net imports for one third of its supplies, Thailand for about two thirds, and Vietnam for about 80% of its supplies. Countries like Vietnam have adopted balanced trade strategies to simultaneously protect the growing domestic dairy farming sector while allowing dairy processors to still access imports to sustain and expand manufacturing of processed products. For example, variable tariff rates involve high tariffs applied on milk powder imports until domestic supplies are exhausted, after which they fall to much lower levels (e.g. 5%).

Finally, Uganda – with its relatively favorable agroecological potential for dairy – is a recent success story driven by growth in domestic production and exports. While its exports reduce total domestic supply, exports offer more scope for sustainable growth of the dairy sector through price stabilization and market growth opportunities (Van Campenhout et al., 2021).

However, we observe several countries in Table 3 with sustained economic growth but only minimal success in increasing dairy supplies. Bangladesh has seen modest growth in dairy production, but its high tariffs (25%) have led to stagnation in import growth, and nutritional adequacy has only risen from 16% to 23% across three decades of development. Ghana saw virtually no growth in its miniscule domestic sector and only modest growth in imports, and adequacy of supply is currently just 8%.

In summary, there are large variations in the extent of success that different countries have had in achieving growth in dairy supplies, as well as variation in the sources of that growth depending on comparative advantage in dairy production and the extent of proactive industrial policies.

### 4.4 Food safety and quality challenges in the dairy sector

Food safety and quality problems are major challenges in LMIC dairy sectors because of dairy’s high potential growth for growth in pathogenic bacteria in poor production, storage, and processing conditions, and because of the introduction of foreign substances into dairy products, including deliberate adulteration. Due to various dairy-related public health disasters in industrializing economies in the late 1800s and early 1900s (Boor et al., 2017, Garcia et al., 2019), regulation of dairy value chains improved. The introduction of milk inspections in the US reduced mortality from waterborne and foodborne diseases by 12–19 percent (Anderson et al., 2022), while the US Department of Agriculture estimated that about 25% of foodborne and waterborne illnesses in the US in 1938 had been caused by consumption of contaminated dairy products, compared to less than 1% today (Boor et al., 2017).

Although these statistics demonstrate that dairy products *can* be made safe, the WHO’s Global Burden of Foodborne Disease project estimated that dairy products were responsible for around 4% of the global foodborne disease burden in 2010, though this may be an underestimate (Grace et al., 2020).^15^ Part of the challenge stems from the high degree of informality in dairy sectors in many LMICs, as well as poor knowledge of health hazards among value chain actors and consumers. However, even in the formal sector in LMICs there is often surprisingly low adoption of hygienic practices and high rates of contamination (Grace et al., 2020). In Ethiopia, Minten et al. (2023) find low adoption of hygienic practices among formal dairy producers, but also that pasteurized milk did not receive any price premium at retail outlets, perhaps suggesting consumer demand for food safety may be weak. In this collection, Janssen and Swinnen (2019) similarly find that access to improved value chains had little impacts on adoption of food safety measures in the Indian context, though another study in this collection conducted in Indonesia found that dairy cooperative members had milk samples with high milk quality and safety (Fadillah et al., 2023). Other studies argue that too many LMIC government strategies focus on formalizing the sector and on punishing or inhibiting informal traders, rather than positive interventions (such as training) that improve storage and handling practices. Blackmore et al. (2022) refer to this as the regulation-reality gap.

In this special collection, Muunda et al. (2021) simulate the potential impacts of introducing new regulations to increase registration and licensing of smallholder producers and dairy business operators, improve product hygiene and quality, and safeguard the health of consumers, including the requirement to pasteurize milk before it is sold and adopt traceability processes and quality tests. They suggest that these regulations would increase retail prices of milk, resulting in decreased milk allocation to and intake by children.

Also in this special collection, Oliveira et al. (2023) analyze how the creation of a private *meso*-institution (*Conseleite*) affected the implementation of food safety guidelines in the Brazilian dairy industry, which has also been affected by numerous food safety scandals in recent decades. Using a difference-in-difference approach on panel data collected over 2006–2014, they show that the creation of *Conseleite* led to a significant decrease in bacterial contamination of milk. The authors argue that food safety *meso*-institutions can play a critical role in bridging the gap between the “institutional environment” level and the “governance” level. In the Chinese context, where the 2008 melamine scandals severely eroded consumer trust in dairy products, though Li et al. (2021) show that consumer trust in the government and third party institutions was still relatively strong. Consistent with that, Jin et al. (2023) empirically examine the role of third-party certification in reducing the negative spillover effects of the crisis, and show that certification helped innocent firms better resist and recover, especially those with lower established reputations.

### 4.5 Gender challenges: The role of women as dairy producers, consumers, and caregivers

In many LMICs many women play an important role in dairy production (Kimaro et al., 2013, Nyongesa et al., 2016) and sometimes in marketing (McPeak and Doss, 2006), while also being critically important purchasers and preparers of dairy dairy-based foods, including for their children.

On the production side, women are often important in providing the labor for taking care of cattle and milking cows, but there are longstanding concerns that in many cultures women are marginalized from decision-making on dairy marketing and investment decisions (Johnson et al., 2015, Tavenner et al., 2018). Such marginalization is by no means universal – in pastoralist Kenya, one study found that women were heavily engaged in milk marketing (McPeak and Doss, 2006) – and unfortunately there are few very specific statistics on women’s marketing activities and retention and control of dairy-related income. In Bangladesh a study by Roy et al. (2015) found that women receiving cow transfers as part of a social protection program retained ownership of the cows, but other assets purchased out of dairy profits were retained by men. There are likewise concerns that female dairy farmers are often marginalized from access to inputs and training, which are sometimes also provided by marketing organizations such as cooperatives and MCCs. Hence there is a need to strengthen the gender inclusion capacities of marketing actors and institutions, both because of the importance of women in farming, food preparation and child-rearing, but also because of the benefits of targeting women as clients of value chain services (Basu et al., 2019, Katothya, 2017).

In India, women are traditionally highly marginalized in a wide range of agricultural activities, including dairy. Studies of Indian dairy cooperatives yield somewhat ambiguous results on their capacity to improve women’s empowerment in dairy value chains. Some reviews conclude that women-only dairy cooperatives may have limited empowerment benefits when they stop short of actively challenging gender and caste norms at the community level (Farnworth et al., 2023b).

Specific studies on the impacts of dairy commercialization on both women’s roles and child nutrition are rare, however. A qualitative study in Kenya found that children from more commercialized dairy households received more milk and their mothers spent less time on dairy activities (Micere Njuki et al., 2016). Results on decision-making were complex however, with women in more commercialized households having more say in decisions but less control over total dairy income. Another study in Kenya found suggestive evidence that when dairy cows are co-owned by women and men there is a stronger positive association with child nutrition outcomes (Jin and Iannotti, 2014).

In this collection, Farnworth et al. (2023a) study gender norms in the context of Rwanda’s nationwide “One Cow per Family” program. Despite impressive accomplishments in economic gender-sensitive interventions in the dairy sector have been quite limited. In their qualitative study, Farnworth et al., 2023a, Farnworth et al., 2023b find that gender norms about “appropriate” masculine and feminine behaviors strongly affect intra-household nutrition, with men normatively responsible for providing money to purchase food and women to buy and prepare food. Yet men reportedly often fail to provide sufficient money for purchasing ASFs or may sell ASFs rather than allocate them to their families. Encouragingly, however, male respondents were strongly interested in being trained on nutrition.

### 4.6 Climate change, conflict, and other challenges to dairy development in LMICs

Climate change is a serious challenge for dairy development in LMICs as well as more temperate high income “dairy frontier” countries. On a purely physiological front, heat stress has adverse effects on productivity, fertility and health in dairy cattle (Habimana et al., 2023). Tropical cattle breeds have greater heat tolerance than temperate breeds, but lower productivity, such that cross-bred cattle have become the dominant source of dairy herds through most LMICs (Godde et al., 2021). However, even more resilient cross-bred cattle may struggle to cope with more frequent extreme weather events.

On a socio-economic level, more unpredictable rainfall is a particularly acute problem for transhumant pastoralists – especially in the Sahel and Horn of Africa – who depend on migration in search of pasture. In a large empirical study on rainfall shocks and violent conflicts in Africa over 1989–2018, McGuirk and Nunn (2020) shows that droughts in the territory of transhumant pastoralists lead to conflict in neighboring agricultural areas, instead of cooperative agreements. An article in this volume by Fadare et al. (2023) finds that exposure to farmer-herder conflict in Nigeria reduces the quantity of ASFs consumed by households, but that livestock diversification has a positive buffering effect. They emphasize the need to implement timely nutrition interventions in conflict-affected areas, and to promote conflict-sensitive interventions.

Finally, production and marketing of animal source foods is a major contributor to Green House Gas (GHG) emissions and other environmental stressors, and may contribute to 2–3% of anthropogenic GHGs (Gerber et al., 2010). That said, dairy production is often inappropriately bundled together with beef production as one of the highest emission sectors, when its nutrient production per GHG unit in dairy is much higher than in beef. Milk is so high in protein that GHG emissions per unit of protein are no higher for dairy than they are for bananas, and just one fifth the level of beef (Poore and Nemecek, 2018).^16^ Quality-adjusted protein or micronutrient calculations would further improve dairy’s nutrient-GHG ratios relative to most other foods.

Even so, there are significant opportunities to reduce the global environmental footprint of dairy through more efficient dairy production systems in LMICs (Tricarico et al., 2020). Existing estimates suggest that dairy herds in Asia, Latin America and Africa account for the bulk of the world’s dairy-related GHG emissions and 3–5 times more GHGs per unit of fat- and protein-adjusted milk because yields are so much lower in these regions (FAO, 2013a), including widespread use of dual purpose breeds rather than specialized dairy animals (Hagemann et al., 2012). Dairy farms in the “frontier” countries, in contrast, have achieved a massive reduction in GHGs because of productivity improvements; Californian dairy farmers cut GHG emissions per unit of milk by 45% from the 1960s to the 2000s. However, scope for that degree of environmental efficiency gain will often be more limited in LMICs because their yield potential is inherently lower. In this virtual collection, Vogel et al. (2023) assess tradeoffs and synergies between production and environmental goals in pasture-based dairy farms in Brazil using stochastic frontier analysis with methane emissions as an undesirable output. They find that farmers can improve their production by 9.4% while simultaneously reducing methane emissions by 8.7% via adoption of more productive cows and improved pastures. That finding is likely context-specific, however, but Vogel et al. (2023) provide a strong method for assessing environmental tradeoffs and synergies in other LMIC settings.

### 4.7 Dairy demand challenges in LMICs

Income elasticities for dairy products are generally high in LMICs, even in countries with relatively high lactose intolerance (Colen et al., 2018). Cross-country differences in average incomes and relative dairy prices explain perhaps three quarters of the difference in dairy consumption prevalence among children 6–23 months of age, although lack of refrigeration also seems to explain low demand in some LMICs (Headey, 2023). In this special collection, Ecker and Pauw (2023) analyze household food consumption data from representative surveys in Kenya and Uganda. Dairy consumption among the richest quintile is three times that of the poorest quintile in Kenya and four times that of the poorest quintile in Uganda. Applying a QUAIDS model to nationally representative household survey data in the two countries, they find that income elasticities for dairy are high in urban areas (around 1.20), but lower in rural areas, especially among the poor (just 0.50 in Uganda and 0.75 in Kenya). The authors also show that household demand for fish and meat are stronger than they are for dairy products.

While we know that demand for dairy products generally rises with household income or wealth in LMICs, we know much less about interventions to stimulate demand, and the synergies between dairy demand and supply interventions. There is one important exception, however: Thailand.

Smitasiri and Chotiboriboon (2003) and Yothasamut et al. (2018) review the history of Thailand’s efforts to stimulate demand for dairy products in a country with no tradition of dairy consumption. With cooperation from the Danish government, Thailand promoted dairy production in its more temperate regions in the 1960s and 1970s. However, by the early 1980s the country’s relatively small dairy sector was producing more milk than it could sell in the domestic market. To simultaneously support dairy farmers, foster lifelong demand for dairy, and to improve child nutrition in the immediate future, The National Milk Drinking Campaign Board (NMDCB) was charged with coordinating a national school milk program in 1992. The program distributes free plain milk to children aged 3–12 years old in all state-owned and some privately owned schools. For most of its history, the school milk program has absorbed 30–40% of Thailand’s domestic dairy production. Although NMDCB played a leading role, they did so with support from the Ministries of Education and of Public Health. In addition to the school milk program, these agencies also supported national education campaigns such as the “Give milk to the one you love” campaign and various national dairy milestone campaigns, though in recent decades private advertising is at least as far-reaching as public campaigns if not more so.

Although it is difficult to assign a counterfactual scenario, Thailand’s efforts at dairy demand promotion have almost certainly been successful. Our rough estimate suggests that the nutritional adequacy of dairy supplies grew from 20% in the early 1990 s to 50% over 2015–2019. Moreover, widespread provision of dairy supplements in schools has almost certainly helped establish a strong dairy culture in Thailand and widespread awareness of dairy as a nutritious child-friendly food. Indeed, Yothasamut et al. (2018) raise concerns that consumer perceptions of dairy as being highly stimulative to child growth has led some Thai caregivers to over-feed milk to their children.

## 5 Conclusions

Dairy has tremendous potential for reducing the global burden of undernutrition and at the same time augmenting the incomes of farm households in a wide range of countries. There are, however, multiple challenges to sustainably and inclusively expanding production and marketing of milk in tropical and increasingly warmer LMIC climates. Moreover, while consumer preferences for milk appear strong, the combination of low incomes, high prices, and limited product availability constitute major constraints on the demand for milk, and countries with weak dairy traditions may also require more proactive efforts to stimulate demand, such as school and pre-school feeding programs.

The studies in this collection of articles and those cited in this review illustrate dairy’s potential, the extent of the consumption gap, and potential strategies for closing that gap. That said, there are many important areas for further research on dairy development for nutrition.

First, while there is diverse but remarkably robust evidence that access to dairy products reduces child stunting and redresses micronutrient deficiencies, more research on cognitive development outcomes is warranted (Lee et al., 2018).

Second, while we have highlighted several recent dairy development success stories in both Africa and Asia (Habiyaremye et al., 2021; Nguyen et al., 2021; Van Campenhout et al., 2021), rigorous evidence that would permit us to ascertain causality rather than association remains limited. Further, there is much to be learned from the diversity of different dairy development initiatives, and the conditions for successful replication or adaptation.

Third, on the demand side, school feeding interventions have been adopted in numerous LMICs, often at scale, but adoption of dairy programs in pre-schools or complementary feeding programs is much rarer in LMICs, despite strong evidence of beneficial impacts. In our view, dairy is a highly under-utilized complementary food, and national nutrition strategies need to attach much greater priority to promoting dairy consumption, while researchers need to study the most effective means of doing so. Demand-focused research also needs to consider ways to reduce consumption of dairy products with added fats and sugars.

Fourth, at a global level, research is needed on the economic, nutritional and environmental impacts of alternative dairy development scenarios. For example, should production be more specialized in temperate areas to reduce environmental footprints, even with GHG emissions from dairy-related transport costs from international trade? Or are there still economic, social and environmental rationales for expanded production of dairy in the tropics?

These are critically important research topics for finding strategies that maximizes dairy’s contribution to redressing undernutrition (and averting obesity) as well as to important economic objectives in LMICs such as poverty reduction, whilst minimizing dairy’s environmental footprint.

## CRediT authorship contribution statement

**Derek D. Headey:** Conceptualization, Investigation, Methodology, Visualization, Writing – original draft, Writing – review & editing. **Harold Alderman:** Conceptualization, Investigation, Methodology, Writing – original draft, Writing – review & editing. **John Hoddinott:** Conceptualization, Investigation, Methodology, Writing – original draft, Writing – review & editing. **Sudha Narayanan:** Investigation, Writing – original draft, Writing – review & editing.

## Declaration of Competing Interest

The authors declare that they have no known competing financial interests or personal relationships that could have appeared to influence the work reported in this paper.

## Footnotes

7 The bulk of milk production takes place in the morning, and in many settings households try to sell much of the morning milk. However, cows typically also express some milk in the evening, which – without access to refrigeration or markets – is often used for household consumption.

## Footnotes

1 A Cochrane review on “Effectiveness of provision of animal‐source foods for supporting optimal growth and development in children 6 to 59 months of age” did not include any studies that provided milk (Eaton et al., 2019).

2 Rawlins et al. (2014) and Kabunga et al. (2017) focus on access to improved dairy breeds while Choudhary and Headey (2018) focus on dairy production in the past 12 months, and Hoddinott et al. (2015) only focus on cow ownership and uncover smaller “impact” estimates of around 0.3 standard deviations instead of 0.5 standard deviations. However, Choudhary and Headey (2018) show that if Hoddinott et al. (2015) focused only on those cows that actually produced milk in the past 12 months, then they would uncover an impact estimate roughly equal to the 0.5 standard deviation estimate found in the other studies mentioned in this paragraph.

3 A skeptic might be concerned that the observational evidence on dairy production/consumption and child HAZ is flawed because dairy production or consumption represents imperfectly observed wealth and the sizable income elasticity of household demand for dairy products. The studies above generally try to address these concerns through placebo tests, but one remarkably consistent result is that the association between HAZ/stunting and non-dairy ASFs is significantly weaker than that of dairy (Haile and Headey, 2023; Zoniaina 2023; Headey et al. 2023; Choudhary and Headey 2018), despite meat, fish and eggs also being foods highly demanded by LMIC households (Colen et al., 2018). Haile and Headey (2023) use a cross-country panel regression with country fixed effects and controls for GDP, consumption of non-dairy foods, and health, education, and sanitation indicators.

4 One review noted that while cross-sectional studies tended to find an inverse relationship between dairy intake and body mass, prospective studies tended to a positive but modest relationship (Babio et al., 2022). Hence, in addition to knowledge gaps on the mechanisms by which dairy intake affect weight gain/loss, there is some inconsistency in conclusions based on the type of analyses conducted.

5 These DHS data do not tell us about quantities consumed, but they are nationally representative, accurate and highly standardized survey instruments. Since other ASFs can offer high quality protein and many of the nutrients provided by dairy (as well as iron, which dairy does not offer), the DHS also allows us to compare consumption of dairy to consumption of other ASFs.

6 It is worth noting that the DHS child dietary diversity data have other limitations. We do not know if all infant formula contains milk powder (though most will) and we do not know if cow’s milk is diluted or contaminated in any way, despite some qualitative evidence that even households themselves may dilute their own milk supplies with water to ensure all household members have milk to drink (Anato et al., 2023). We also do not know if some dairy products have added sugar.

8 On the extensive margin, cattle ownership does not provide specific information on dairy-producing cattle specifically, but it does illustrate broad constraints on cattle rearing, particularly the strong association between cattle ownership and climate conditions.

9 Yield estimates for low- and middle-income countries in the FAOSTAT are more speculative because of the lower degree of commercialization in the sector and weaker statistical systems, and the fact that cattle populations are often used for dairy and other agricultural and transport purposes. Still, national official estimates for Kenya are likely relatively accurate, and the basic conclusion that yields in many LMICs are just a fraction of those in the dairy frontier countries is surely robust.

10 Indeed, the world’s largest dairy exporter – New Zealand’s Fonterra - is still a cooperative with around 9000 farmers, and accounts for 30% of the world’s milk exports.

11 FDI played a crucial role in modernizing the dairy sectors of Eastern Europe in the 1990s after the end of communism (Dries and Swinnen, 2004; Gorton and Guba, 2002). Its role in the development of East Asia’s dairy sector is largely unstudied.

12 Of course, other dairy products like condensed milk or long-life milk are also non-perishable and tradable, but often costly to import long distances, and relatively unaffordable for many consumers.

13 The persistence of trade barriers even when domestic supplies are insufficient is not nutritionally rational, but the political economy explanation is well known: dairy producers have strong incentives to lobby for protection, while consumers may hardly be aware of the higher costs of dairy products due to trade barriers (Anderson, 2008).

14 We acknowledge both the limitations of these aggregate supply data and the conceptual caveats around “nutritional adequacy”, especially given widespread lactose intolerance.

15 Harmful bacteria may originate from the animal, the environment, milking equipment, or the milk handlers or be introduced with an adulterant such as contaminated water. Chemical contaminants may enter the milk through the feed or veterinary treatments of the animal or through later accidental or deliberate contamination Grace et al. (2020).

16 See also: https://ourworldindata.org/environmental-impacts-of-food.

## References

[b0005] Alderman, H., 1987. Cooperative dairy development in Karnataka, India: an assessment, Research report No. 64. International Food Policy Research Institute (IFPRI), Washington DC. http://ideas.repec.org/p/fpr/resrep/64.html.

[b0010] Alsan M. (2015). The effect of the TseTse fly on African development. Am. Econ. Rev..

[b0015] Anato, A., Headey, D., Hirvonen, K., Pokharel, A., Tessema, M., We, F., Baye, K. 2023. From animal feed to milk consumption: assessment of the risk of aflatoxin contamination, IFPRI Discussion Paper. Washington DC.

[b0020] Anderson K. (2008).

[b0025] Anderson, D.M., Charles, K.K., McKelligott, M., Rees, D.I.. 2022. Safeguarding Consumers Through Minimum Quality Standards: Milk Inspections and Urban Mortality, 1880-1910. National Bureau of Economic Research Working Paper Series No. 30063.

[b0030] Ava J. (2014).

[b0035] Babio N., Becerra-Tomás N., Nishi S.K., López-González L., Paz-Graniel I., García-Gavilán J., Schröder H., Martín-Calvo N., Salas-Salvadó J. (2022). Total dairy consumption in relation to overweight and obesity in children and adolescents: A systematic review and meta-analysis. Obes. Rev..

[b0040] Basu P., Galiè A., Baltenweck I. (2019). Presence and property: Gendered perspectives on participation in a dairy development program in Kenya and Uganda. Women's Stud. Int. Forum.

[b0045] Behnke, R., 2010. The Contribution of Livestock to the Economies of IGAD Member States: Study Findings, Application of the Methodology in Ethiopia and Recommendations for Further Work, IGAD LPI Working Paper No. 02 - 10. Intergovernmental Authority on Develpoment, Addis Ababa. http://www.igad-data.org/index.php?option=com_docman&Itemid=42.

[b0050] Black R.E., Victora C.G., Walker S.P., Bhutta Z.A., Christian P., de Onis M., Ezzati M., Grantham-McGregor S., Katz J., Martorell R., Uauy R. (2013). Maternal and child undernutrition and overweight in low-income and middle-income countries. Lancet.

[b0055] Blackmore E., Guarin A., Vorley W., Alonso S., Grace D. (2022). Kenya’s informal milk markets and the regulation–reality gap. Dev Policy Rev..

[b0060] Boor K.J., Wiedmann M., Murphy S., Alcaine S. (2017). A 100-year review: microbiology and safety of milk handling. J. Dairy Sci..

[b0065] Burkitbayeva S., Janssen E., Swinnen J. (2023). Hiding in plain sight: the emergence of modern dairy farms in India. J. Agribus. Dev. Emerg. Econ..

[b0070] Candler W., Kumar N. (1998).

[b0075] Chagwiza C., Muradian R., Ruben R. (2016). Cooperative membership and dairy performance among smallholders in Ethiopia. Food Policy.

[b0080] Choudhury S., Headey D.D. (2018). Household dairy production and child growth: evidence from Bangladesh. Econ. Hum. Biol..

[b0085] Colen L., Melo P.C., Abdul-Salam Y., Roberts D., Mary S., Gomez Y., Paloma S. (2018). Income elasticities for food, calories and nutrients across Africa: a meta-analysis. Food Policy.

[b0090] de Beer H. (2012). Dairy products and physical stature: a systematic review and meta-analysis of controlled trials. Econ. Hum. Biol..

[b0095] de Lamas C., de Castro M.J., Gil-Campos M., Gil Á., Couce M.L., Leis R. (2019). Effects of dairy product consumption on height and bone mineral content in children: a systematic review of controlled trials. Adv. Nutr..

[b0100] De Vries A., Kaylegian K.E., Dahl G.E. (2020). MILK Symposium review: Improving the productivity, quality, and safety of milk in Rwanda and Nepal*. J. Dairy Sci..

[b0105] Dervillé M., Manriquez D., Dorin B., Aubron C., Raboisson D. (2023). Indian dairy cooperative development: a combination of scaling up and scaling out producing a center-periphery structure. World Dev..

[b0110] Dougkas A., Barr S., Reddy S., Summerbell C.D. (2019). A critical review of the role of milk and other dairy products in the development of obesity in children and adolescents. Nutr. Res. Rev..

[b0115] Dries L., Swinnen J.F.M. (2004). Foreign direct investment, vertical integration, and local suppliers: evidence from the polish dairy sector. World Dev..

[b0120] Dyer A.H., Vahdatpour C., Sanfeliu A., Tropea D. (2016). The role of Insulin-Like Growth Factor 1 (IGF-1) in brain development, maturation and neuroplasticity. Neuroscience.

[b0125] Eaton J.C., Rothpletz-Puglia P., Dreker M.R., Iannotti L., Lutter C., Kaganda J., Rayco-Solon P. (2019). Effectiveness of provision of animal-source foods for supporting optimal growth and development in children 6 to 59 months of age. Cochrane Database Syst. Rev..

[b0130] Ecker, O., Pauw, K., 2023. Dairy Consumption and Household Diet Quality in East Africa: Evidence from Survey-Based Simulation Models. Food Policy.

[b0135] Fadare, O., Fadare, O.o.A.O., Srinivasan, C., Zanello, G., 2023. Livestock Diversification Mitigates the Impact of Farmer-Herder Conflict on Animal-Source Foods Consumption in Nigeria, Fadare et al. Food Policy.

[b0140] Fadillah A., van den Borne B.H.P., Poetri O.N., Hogeveen H., Umberger W., Hetherington J., Schukken Y.H. (2023). Smallholder milk-quality awareness in Indonesian dairy farms. J. Dairy Sci..

[b0145] Fao (2008).

[b0150] FAO (2013).

[b0155] FAO (2013).

[b0160] FAO, 2023. FAOSTAT. Food and Agriculture Organization, Rome.

[b0165] FAO, GDP, & IFCN. 2018. Dairy Development's Impact on Poverty Reduction. Food and Agriculture Organization, Global Dairy Platform, and IFCN Dairy Research Network, Rome. http://www.fao.org/3/CA0289EN/ca0289en.pdf.

[b0170] FAO/WHO/UNU, 2007. Protein and amino acid requirements in human nutrition, WHO Technical Report Series 935. World Health Organisation, Geneva. 18330140

[b0175] Farnworth C.R., Jumba H., Otieno P.E., Galiè A., Ouma E., Flax V.L., Schreiner M.-A., Colverson K. (2023). Gender roles and masculinities in leveraging milk for household nutrition: Evidence from two districts in Rwanda. Food Policy.

[b0180] Farnworth C.R., Ravichandran T., Galiè A. (2023). Empowering women across gender and caste in a women’s dairy cooperative in India. Front. Sustain. Food Syst..

[b0185] Garcia S.N., Osburn B.I., Cullor J.S. (2019). A one health perspective on dairy production and dairy food safety. One Health.

[b0190] Gatica-Domínguez G., Neves P.A.R., Barros A.J.D., Victora C.G. (2021). Complementary feeding practices in 80 low- and middle-income countries: prevalence of and socioeconomic inequalities in dietary diversity, meal frequency, and dietary adequacy. J. Nutr..

[b0195] Gebreyohanes G., Yilma Z., Moyo S., Mwai O.A. (2021).

[b0200] Geng X., Janssens W., Kramer B. (2023). Liquid milk: Savings, insurance and side-selling in cooperatives. J. Dev. Econ..

[b0205] Gerber, P., Vellinga, T., Dietze, K., Falcucci, A., Gianni, G., Mounsey, J., Maiorano, L., Opio, C., Sironi, D., Thieme, O., Weiler, V., 2010. Greenhouse Gas Emissions from the Dairy Sector: A Life Cycle Assessment. Food and Agriculture Organization, Rome. https://www.fao.org/3/k7930e/k7930e00.pdf.

[b0210] Godde C.M., Mason-D’Croz D., Mayberry D.E., Thornton P.K., Herrero M. (2021). Impacts of climate change on the livestock food supply chain; a review of the evidence. Glob. Food Sec..

[b0215] Gorton M., Guba F. (2002). Foreign Direct Investment (FDI) and the restructuring of the Hungarian dairy processing sector. J. East-West Bus..

[b0220] Grace D., Wu F., Havelaar A.H. (2020). MILK Symposium review: Foodborne diseases from milk and milk products in developing countries—Review of causes and health and economic implications*. J. Dairy Sci..

[b0225] Grenov B., Michaelsen K.F. (2018). Growth components of cow's milk: emphasis on effects in undernourished children. Food Nutr. Bull..

[b0230] Habimana V., Nguluma A.S., Nziku Z.C., Ekine-Dzivenu C.C., Morota G., Mrode R., Chenyambuga S.W. (2023). Heat stress effects on milk yield traits and metabolites and mitigation strategies for dairy cattle breeds reared in tropical and sub-tropical countries. Front. Vet. Sci..

[b0235] Habiyaremye N., Ouma E.A., Mtimet N., Obare G.A. (2021). A review of the evolution of dairy policies and regulations in Rwanda and its implications on inputs and services delivery. Front. Vet. Sci..

[b0240] Habiyaremye N., Mtimet N., Ouma E.A., Obare G.A. (2023). Cooperative membership effects on farmers’ choice of milk marketing channels in Rwanda. Food Policy.

[b0245] Hagemann M., Ndambi A., Hemme T., Latacz-Lohmann U. (2012). Contribution of milk production to global greenhouse gas emissions. Environ. Sci. Pollut. Res..

[b0250] Haile B., Headey D. (2023). Growth in milk consumption and reductions in child stunting: historical evidence from cross-country panel data. Food Policy.

[b0255] Headey D. (2023). Can dairy help solve the malnutrition crisis in developing countries? An economic analysis. Anim. Front..

[b0260] Headey D.D., Alderman H.H. (2019). The relative caloric prices of healthy and unhealthy foods differ systematically across income levels and continents. J. Nutr..

[b0265] Headey D., Hirvonen K., Hoddinott J. (2018). Animal sourced foods and child stunting. Am. J. Agric. Econ..

[b0270] Headey D.D., Palloni G. (2020). Stunting and wasting among Indian preschoolers have moderate but significant associations with the vegetarian status of their mothers. J. Nutr..

[b0275] Heard B.R., Thi H.T., Burra D.D., Heller M.C., Miller S.A., Duong T.T., Simioni M., Jones A.D. (2020). The influence of household refrigerator ownership on diets in Vietnam. Econ. Hum. Biol..

[b0280] Herber C., Bogler L., Subramanian S.V., Vollmer S. (2020). Association between milk consumption and child growth for children aged 6–59 months. Sci. Rep..

[b0285] Herrero, M., Di Mayberry, D., van de Steeg, J., Phelan, D., Ash, A., Diyezee, K., Robinson, T., Henderson, B., Gilbert, M., van Wijk, M., Godde, C., Blummel, M., Prestwidge, D., Stephenson, E., Power, B., Parsons, D.T., 2016. Understanding livestock yield gaps for poverty alleviation, food security and the environment, The LivesGAPS project - final report. Commonwealth Scientific & Industrial Research Organisation (CSIRO), Brisbane. https://cgspace.cgiar.org/handle/10568/93009.

[b0290] Hoddinott J., Behrman J.R., Maluccio J.A., Melgar P., Quisumbing A.R., Ramirez-Zea M., Stein A.D., Yount K.M., Martorell R. (2013). Adult consequences of growth failure in early childhood. Am. J. Clin. Nutr..

[b0295] Hoddinott J., Headey D., Dereje M. (2015). Cows, missing milk markets, and nutrition in rural Ethiopia. J. Dev. Stud..

[b0300] Huang M.N., Headey D., Nguyen P. (2023).

[b0305] Iannotti L., Muehlhoff E., McMahon D. (2013). Review of milk and dairy programmes affecting nutrition. J. Dev. Eff..

[b0310] ICF International, 2022. The Demographic and Health Surveys Program. ICF International, Calverton MD.

[b0315] Janssen E., Swinnen J. (2019). Technology adoption and value chains in developing countries: Evidence from dairy in India. Food Policy.

[b0320] Jin M., Iannotti L.L. (2014). Livestock production, animal source food intake, and young child growth: the role of gender for ensuring nutrition impacts. Soc. Sci. Med..

[b0325] Jin Y., Zhou J., Ye J. (2023). Value of certification in collective reputation crises: evidence from Chinese dairy firms. Food Policy.

[b0330] Johnson N., Njuki J., Waithanji E., Nhambeto M., Rogers M., Kruger E.H. (2015). The gendered impacts of agricultural asset transfer projects: lessons from the Manica smallholder dairy development program. Gend. Technol. Dev..

[b0335] Kabunga N.S., Ghosh S., Webb P. (2017). Does ownership of improved dairy cow breeds improve child nutrition? A pathway analysis for Uganda. PLoS One.

[b0340] Kang K., Sotunde O.F., Weiler H.A. (2019). Effects of milk and milk-product consumption on growth among children and adolescents aged 6–18 years: a meta-analysis of randomized controlled trials. Adv. Nutr..

[b0345] Katothya G. (2017).

[b0350] Kimaro E.G., Lyimo-Macha J.G., Jeckoniah J.N. (2013). Gender roles in small holder dairy farming: pertinent issues on access and control over dairy farming resources in Arumeru district, Tanzania. Livest. Res. Rural. Dev..

[b0355] Kondaridze M., Luckstead J. (2023). Determinants of dairy-product trade: do subsidies matter?. J. Agric. Econ..

[b0360] Kumar A., Saroj S., Joshi P.K., Takeshima H. (2018). Does cooperative membership improve household welfare? Evidence from a panel data analysis of smallholder dairy farmers in Bihar, India. Food Policy.

[b0365] Kumar A., Mishra A.K., Saroj S., Joshi P.K. (2019). Impact of traditional versus modern dairy value chains on food security: evidence from India’s dairy sector. Food Policy.

[b0370] Kumar C., Rana R.K., Kumar M., Kujur A., Kashyap V., Singh S.B., Sagar V., Kumari N., Kumar D. (2021). Effect of milk supplementation on the status of micronutrients among rural school children aged 5–19 years in a tribal predominating district of India. BMJ Nutr. Prev. Health.

[b0375] Lee R., Singh L., van Liefde D., Callaghan-Gillespie M., Steiner-Asiedu M., Saalia K., Edwards C., Serena A., Hershey T., Manary M.J. (2018). Milk powder added to a school meal increases cognitive test scores in Ghanaian children. J. Nutr..

[b0380] Leighton, G., Clark, M.L., 1929. Milk consumption and the growth of school children: Second preliminary report on tests to the Scottish Board of Health 1, 23-25. 10.1136/bmj.1.3548.23 PMC2449796 20774373

[b0385] Li S., Wang Y., Tacken G.M.L., Liu Y., Sijtsema S.J. (2021). Consumer trust in the dairy value chain in China: The role of trustworthiness, the melamine scandal, and the media. J. Dairy Sci..

[b0390] Liu X., Liang Y., Kevin Chen P.D. (2023). Dairy trade liberalization and child stunting: evidence from low- and middle-income countries. Food Policy.

[b0395] McGill C.R., Fulgoni V.L., DiRienzo D., Huth P.J., Kurilich A.C., Miller G.D. (2008). Contribution of dairy products to dietary potassium intake in the United States population. J. Am. Coll. Nutr..

[b0400] McGuirk, E.M., Nunn, N., 2020. Transhumant Pastoralism, Climate Change, and Conflict in Africa, NBER Working Paper 28243. National Bureau of Economic Research: Cambridge MA. https://ideas.repec.org/p/nbr/nberwo/28243.html.

[b0405] McPeak J.G., Doss C.R. (2006). Are household production decisions cooperative? Evidence on pastoral migration and milk sales from Northern Kenya. Am. J. Agric. Econ..

[b0410] Micere Njuki J., Wyatt A., Baltenweck I., Yount K., Null C., Ramakrishnan U., Webb Girard A., Sreenath S. (2016). An exploratory study of dairying intensification, women’s decision making, and time use and implications for child nutrition in Kenya. Eur. J. Dev. Res..

[b0415] Michaelsen K.F. (2013). Cow’s milk in the prevention and treatment of stunting and wasting. Food Nutr. Bull..

[b0420] Minten B., Habte Y., Tamru S., Tesfaye A. (2020). The transforming dairy sector in Ethiopia. PLoS One.

[b0425] Minten B., Habte Y., Baye K., Tamru S. (2023). Food safety and incipient modern value chains: evidence from milk in Ethiopia. Eur. J. Dev. Res..

[b0430] Moore L.L., Singer M.R., Qureshi M.M., Bradlee M.L., Daniels S.R. (2012). Food group intake and micronutrient adequacy in adolescent girls. Nutrients.

[b0435] Muunda E., Mtimet N., Schneider F., Wanyoike F., Dominguez-Salas P., Alonso S. (2021). Could the new dairy policy affect milk allocation to infants in Kenya? A best-worst scaling approach. Food Policy.

[b0440] Naik S., Bhide V., Babhulkar A., Mahalle N., Parab S., Thakre R., Kulkarni M. (2013). Daily milk intake improves vitamin B-12 status in young vegetarian Indians: an intervention trial. Nutr. J..

[b0445] Narayanan S., Negi D.S., Gupta T. (2023). Separability, spillovers, and segmented markets: evidence from dairy in India. Agric. Econ..

[b0450] Narrod C., Tiongco M., Costales A., Theime O., Pilling D. (2007). Poultry in the 21st Century: Avian Influenza and Beyond.

[b0455] Nyongesa D., Mwirigi M.K., Yongo D., Makokha S. (2016). Gender-concerns: do they matter in smallholder dairy groups in Kenya?. Int. J. Agric. Resour. Gov. Ecol..

[b0460] Oliveira G.M.d., Miranda B.V., Saes M.S.M., Martino G. (2023). Opening the “black box” of food safety policy implementation: the efficiency-enhancing role of a private meso-institution. Food Policy.

[b0465] Pingali P., Bigot Y., Binswanger H. (1987).

[b0470] Poore J., Nemecek T. (2018). Reducing food’s environmental impacts through producers and consumers. Science.

[b0475] Rawlins R., Pimkina S., Barrett C.B., Pedersen S., Wydick B. (2014). Got milk? The impact of Heifer International’s livestock donation programs in Rwanda on nutritional outcomes. Food Policy.

[b0480] Rizzoli R. (2022). Dairy products and bone health. Aging Clin. Exp. Res..

[b0485] Robinson, T., Thornton, P., Franceschini, G., Kruska, R., Chiozza, F., Notenbaert, A., Cecchi, G., Herrero, M., Epprecht, M., Fritz, S., Liang, Y., Conchedda, G., See, L., You, L., 2011. Global livestock production systems. Food and Agriculture Organization of the United Nations (FAO).

[b0490] Roy S., Ara J., Das N., Quisumbing A.R. (2015). “Flypaper effects” in transfers targeted to women: evidence from BRAC's “Targeting the Ultra Poor” program in Bangladesh. J. Dev. Econ..

[b0495] Saha Turna N., Havelaar A., Adesogan A., Wu F. (2022). Aflatoxin M1 in milk does not contribute substantially to global liver cancer incidence. Am. J. Clin. Nutr..

[b0500] Sibhatu K.T., Qaim M. (2017). Rural food security, subsistence agriculture, and seasonality. PLoS One.

[b0505] Smitasiri S., Chotiboriboon S. (2003). Experience with programs to increase animal source food intake in Thailand. J. Nutr..

[b0510] Snijder M.B., van der Heijden A.A., van Dam R.M., Stehouwer C.D., Hiddink G.J., Nijpels G., Heine R.J., Bouter L.M., Dekker J.M. (2007). Is higher dairy consumption associated with lower body weight and fewer metabolic disturbances? The Hoorn Study. Am. J. Clin. Nutr..

[b0515] Sunardi D., Wibowo Y., Mak T.N., Wang D. (2022). Energy and nutrient intake status among Indonesia children aged 1–5 years with different dairy food consumption patterns. Curr. Dev. Nutr..

[b0520] Tavenner K., Fraval S., Omondi I., Crane T.A. (2018). Gendered reporting of household dynamics in the Kenyan dairy sector: trends and implications for low emissions dairy development. Gend. Technol. Dev..

[b0525] Thorning T.K., Raben A., Tholstrup T., Soedamah-Muthu S.S., Givens I., Astrup A. (2016). Milk and dairy products: good or bad for human health? An assessment of the totality of scientific evidence. Food Nutr. Res..

[b0530] Thornton P.K. (2010). Livestock production: recent trends, future prospects. Philos. Trans. R. Soc., B.

[b0535] Tricarico J.M., Kebreab E., Wattiaux M.A. (2020). MILK Symposium review: Sustainability of dairy production and consumption in low-income countries with emphasis on productivity and environmental impact. J. Dairy Sci..

[b0540] UNICEF (2022).

[b0545] Van Campenhout B., Minten B., Swinnen J.F.M. (2021). Leading the way – foreign direct investment and dairy value chain upgrading in Uganda. Agric. Econ..

[b0550] van den Heuvel E.G.H.M., Steijns J.M.J.M. (2018). Dairy products and bone health: how strong is the scientific evidence?. Nutr. Res. Rev..

[b0555] Vandercasteelen J., Minten B., Tamru S. (2021). Urban proximity, access to value chains, and dairy productivity in Ethiopia. Agric. Econ..

[b0560] Visioli F., Strata A. (2014). Milk, dairy products, and their functional effects in humans: a narrative review of recent evidence. Adv. Nutr. Int. Rev. J..

[b0565] Vogel E., Dalheimer B., Beber C.L., Mori C.d., Palhares J.C.P., Novo A.L.M. (2023). Environmental efficiency and methane abatement costs of dairy farms from Minas Gerais, Brazil. Food Policy.

[b0570] Wallace T.C., Bailey R.L., Lappe J., O'Brien K.O., Wang D.D., Sahni S., Weaver C.M. (2021). Dairy intake and bone health across the lifespan: a systematic review and expert narrative. Crit. Rev. Food Sci. Nutr..

[b0575] Wang J., Chen M., Klein P.G. (2015). China's Dairy United: a new model for milk production. Am. J. Agric. Econ..

[b0580] Weinberg L.G., Berner L.A., Groves J.E. (2004). Nutrient contributions of dairy foods in the United States, Continuing Survey of Food Intakes by Individuals, 1994–1996, 1998. J. Am. Diet. Assoc..

[b0585] Willett, W., Rockström, J., Loken, B., Springmann, M., Lang, T., Vermeulen, S., Garnett, T., Tilman, D., DeClerck, F., Wood, A., Jonell, M., Clark, M., Gordon, L.J., Fanzo, J., Hawkes, C., Zurayk, R., Rivera, J.A., Vries, W.D., Sibanda, L.M., Afshin, A., Chaudhary, A., Herrero, M., Agustina, R., Branca, F., Lartey, A., Fan, S., Crona, B., Fox, E., Bignet, V., Troell, M., Lindahl, T., Singh, S., Cornell, S.E., Reddy, K.S., Narain, S., Nishtar, S., Murray, C.J.L., 2019. Food in the Anthropocene: the EAT–Lancet Commission on healthy diets from sustainable food systems. The Lancet Published Online January 16, 2019. pp. 1-47. doi: 10.1016/S0140-6736(18)31788-4. 10.1016/S0140-6736(18)31788-4 30660336

[b0590] Yothasamut J., Camfield L., Pfeil M. (2018). Practices and values regarding milk consumption among pre-schoolers in Bangkok. Int. J. Qual. Stud. Health Well Being.

[b0595] Zemel M.B. (2004). Role of calcium and dairy products in energy partitioning and weight management. Am. J. Clin. Nutr..

[b0600] Zoniaina R.S., Shiratori S., Rafalimanantsoa J., Sakurai T. (2023). Animal-sourced foods production and early childhood nutrition: panel data evidence in central Madagascar. Food Policy.

